# Assisted reproduction in Spain, outcome and socioeconomic determinants of access

**DOI:** 10.1186/s12939-021-01438-x

**Published:** 2021-07-06

**Authors:** Ido Alon, Jaime Pinilla

**Affiliations:** 1 Department of Development Economics, Research Group on Economics and Management of Innovation, Autonomous University of Madrid, Madrid, Spain; 2 Department of Quantitative Methods in Economics, University of Las Palmas de Gran Canaria, Las Palmas, Spain

**Keywords:** Assisted reproductive technology, Bivariate Probit, Competing risk survival analysis, Inequality, Public coverage, Socioeconomic determinants

## Abstract

**Research question:**

We analyzed two questions. First, the effectiveness of public Assisted Reproductive Technologies (ART) in Spain compared with private ones, measured by the time since initiating ART treatment until achieving pregnancy, accounting for age and socioeconomic factors. Second, socioeconomic determinants of access to ART, referring primarily to financial means derived by employment, income, and wealth.

**Design:**

We applied statistical models on data extracted from the national Spanish Fertility Survey from 2018. The first topic was analyzed by competing risk survival analysis conducted on a sample of 667 women who initiate ART treatment since 2000. The second, by a Bivariate Probit model conducted on a sample of 672 women older than 41 years who required ART services.

**Results:**

The first analysis raised that throughout the treatment, patients treated exclusively in private clinics had on average a higher cumulative incidence of becoming pregnant compared with patients who approached public clinics. The second analysis raised that both higher household equivalent income and higher education increase the likelihood of accessing ART in a private clinic and decrease the tendency of accessing public clinics or failing to access any service. Moreover, being single decreases the likelihood of accessing public clinics or ART services in general.

**Conclusions:**

Long waiting periods could be the main reason for the lower incidence of getting pregnant in public healthcare, explaining why patients choose private over public care. We develop a broader discussion over the extent of Spanish public funding of ART, the unequal medical outcome, and potential options for optimization.

## Key points

In Spain, patients treated exclusively in private clinics had on average a higher cumulative incidence of becoming pregnant compared with patients who approached public clinics.

Higher income and higher education increase the likelihood of accessing ART in a private clinic and decrease the likelihood of accessing public clinics or failing to access any service.

Considering long waiting lists and limited resources in public healthcare, policymakers should analyze resource allocation optimization in ART, focusing on public clinics’ efficacy by reconsidering both the age limit and the number of cycles.

## Introduction

Since 2008, the volume of Assisted Reproductive Technologies (ART) in Spain has increased by nearly 50%, reaching 149,337 In-Vitro Fertilization (IVF) and 34,100 Intrauterine Insemination (IUI) cycles in 2018 [51, 53]. Spain is the largest European ART provider and fourth globally [7, 14, 21, 33].

Given that 10–15% of the entire population may suffer from infertility [1, 5], the rise in ART practices can be explained by several factors. ART is becoming more available, affordable, and endurable while providing better outcomes. Additionally, parenthood postponement increases age-related infertility and upsurges demand [3].

In recent years, Spain enjoyed a sharp increase in the number and quality of ART clinics [51, 53], of which some have become world-leading ART corporations. Nevertheless, Spain has the OECD’s second-highest average maternal age at childbirth (32.1 years in 2017) [43], and 1.3 children per woman, among the lowest in the world. For a few decades, it has been facing elevated unemployment rates among youngsters and gender-gaps concerning employment, salary, and work contracts. Spaniards are among the oldest in Europe to leave parents’ home [38, 43]. These conditions have ongoingly contributed to marriage^1^ and pregnancy delay [2, 11, 18, 38]. Spain has the highest proportion in Europe of first births to mothers aged 40 or above (8.8% of total births in 2017) [22]. In 2018, patients older than 40 years received more than 30% of ART cycles, while 57% of these included egg donations. Spain produces more than half the continent’s egg donations [21, 53].

Regulations and public provision largely determine whether the demand for ART is being met [16, 46]. The Spanish law 14/2006^2^ on ART is among the most liberals in Europe. It enables practices with no defined age limit and regulates fertility preservation [4, 12]. A liberal approach towards gamete donations and Preimplantation Genetic Testing (PGT), and an abundant supply of specialized clinics make Spain one of the world’s most popular destinations for cross-border reproductive care, accounting for about 11% of the country’s ART cycles [50, 53].

ART is an expensive and repetitive treatment with uncertain costs and outcomes. In 2018, average success rates in both public and private clinics in Spain were 8–27% for a regular ART cycle, depends on patient’s age, and around 37% for donor-eggs cycles [53]. Costs in private clinics were 700–1000 Euro for an IUI cycle, 3500–5500 Euro for an IVF cycle, up to 9000 Euros for an egg donation, and up to 10,000 Euro for PGT [28], while public clinics offer treatments free of charge, usually not including gamete donations or PGT.

Cost reduction of ART through public funding is often justified by the demographic implications of infertility and its strong impact on the quality of life, not only from a social perspective but also as a source for depression, anxiety and conflict in relationships [19, 32, 41, 45]. Fauser et al. [23] identified positive attitudes among western European respondents towards some extent of public ART coverage for primary infertility. Additionally, Vida ([57], p. 1) argued that “infertility qualifies as an unpredictable incident against which rational agents would choose to insure under ideal conditions and that ART is thereby a matter of collective responsibility”. Chambers et al. [15] found a positive association between affordability and utilization. Nevertheless, public coverage is important not only to provide equal access but also to reduce the financial burden on young parents [16]. It also serves to moderate the principal-agent problem, in which physicians may potentially lead to commodification and supplier-induced demand by offering unnecessary treatments and add-ons [4, 17, 27, 44].

Spanish national health system covers up to three ART cycles for childless couples, up to 40 years for women and 55 for men [4]. However, patients spend around one year on average on waiting lists for public clinics [25, 40], which may delay the potential solution, harm treatment outcomes [13], and produce distrust. In practice, about 75% of cycles are elaborated by private clinics, which are also responsible for nearly all donor eggs^3^ and PGT (therefore for the majority of reproductive tourism) [42, 48, 53]. Some private insurance policies cover a limited number of cycles, usually one or two, with few exceptions. Coverage for donor gametes and PGT is limited.

This study examines two topics. First, the effectiveness of public ART services in Spain compared with private services, measured by the time since initiating treatment until achieving pregnancy, accounting for age and some socioeconomic factors. Second, the socio economic determinants of access to ART, referring primarily to financial means derived by employment, income, and wealth. Additionally, education levels are usually associated with both the postponement of childbearing and a larger tendency to seek medical help. Lower socioeconomic background (and education) may be associated with higher infertility levels, while a higher socioeconomic background may be associated with higher tendency to seek medical solutions [9, 19, 20].

This study supplements previous research about diverse public ART policies concerning the balance between public and private care, socioeconomic disparities, and differences in outcome [12, 13, 54]. We applied two statistical models on data extracted from the national Spanish Fertility Survey (SFS), conducted in 2018 by the National Statistics Institute (INE). First, competing risk survival analysis with ‘stopping the treatment’ as the competing event. Second, a Bivariate Probit (Biprobit) model to identify how socioeconomic determinants affect access to services in public and private clinics.

The first model suggested that patients treated exclusively in private clinics had a higher cumulative incidence of becoming pregnant, on average and throughout the treatment, compared with patients who approached public clinics. The second showed evidence for inequality in access based on income, education levels, and marital status. Additionally, it shows that many patients choose private over public. Finally, we developed a broader discussion over the extent of public funding of ART, long waiting periods for public care, unequal medical outcome, and optimization.

## Materials and methods

Our data source is the SFS [31], which addressed participants aged 18 to 55 who reside in family households throughout the Spanish national territory. It was conducted to identify the determining factors of recent, current, and expected fertility, i.e., factors influencing whether or not (and when) to have children. The SFS was household-based with stratified two-stage sampling and included 17,175 participants (14,556 women and 2619 men).^4^

### Variable definition

Two questions are elaborated in this study: 1) Do public ART services in Spain provide an effective solution compared to private services? 2) How socioeconomic determinants affect access to ART in public and private clinics? As shown in Table 1, our samples comprise 1) women who reported to undergo ART (including IUI) since 2000 or are currently undergoing treatment. 2) Women aged 41 years or more who required ART services.

For both samples, timed intercourse and surrogate gestation have not been considered. In the first, access to ART should have been carried out by the year 2000 or later to avoid the bias of available technology. Concerning the second, women younger than 41 were not included since they may still access public coverage in the future, which may bias our results. To enable our analysis of socioeconomic determinants, some respondents were excluded. Such are those whose reasons not to access ART were: ‘preference for adoption or surrogacy’, ‘physical and health impediments’, ‘religious impediments’ and ‘unwillingness by the partner’, since it is unclear what stands behind their unwillingness (e.g., financial, religion or any other reasons). Conversely, we included the reasons ‘Financial barriers’ (83 respondents), ‘lack of coverage by social security’ (35), ‘lack of time’ (17), and ‘fear of stress or emotional burden’ (33). These reasons may very well be associated with socioeconomics determinants since a favorable financial situation may “buy” time and reduce stress, although demanding professional career produces “lack of time”.

**Table 1:** Inclusion and exclusion criteria for each study question

Question 1: Do public ART clinics in Spain provide an effective solution compared to private ART clinics?	Question 2: How socioeconomic determinants affect access to ART in public and private clinics in Spain?
**Inclusion criteria:** Women who initiate ART treatment since 2000. *N* = 690	**Inclusion criteria:** Women aged 41 or older who tried to get pregnant. *N* = 5897
**Exclusion criteria:** Type of ART timed intercourse (22) or surrogate gestation (1). *N* = 23	**Exclusion criteria (**Reasons for non-access to ART)**:** Non-requirement (5110), physical and health impediments (59), preference for adoption or surrogacy (13), religious impediments (10) and unwillingness by the partner (22), timed intercourse was applied (11). *N* = 5225
Final Sample size = 667	Final Sample size = 672

Our analysis focused on several outcomes related to access to ART. The socioeconomics determinants of access to treatment included in our regression models are marital status,^5^ education level, equivalent monthly household^6^ and personal income in euros, and geographic location (autonomous community).

### Statistical analysis

Our two study questions were answered using the following statistical approaches: First, competing risk survival analysis with ‘stopping the treatment’ as the competing event. This model serves to identify factors associated with the success of ART. Second, a Bivariate Probit (Biprobit) model to study how socioeconomic determinants affect access to ART in public and private clinics.

The datasets included sample weights to account for sample balancing. The standard errors associated with specific estimates, *p*-values and confidence intervals are adjusted for the design effects.

### Competing risk regression

Cross-sectional SFS data in 2018 were used as baseline time-points for a monthly backward tracing of outcomes related to ART based on respondents’ answers about their past and present experiences. The SFS provides data from 667 women who underwent ART in Spain between 2000 and 2018. The data includes the number of treatment duration in months. The end of the treatment was reported as two different competing events: Success, i.e., ‘I have had a child, or I am pregnant’, and ‘stopped treatment’. Observations were censored if the person is currently being treated.

We are interested in studying the duration from initiating ART treatment until achieving pregnancy concerning age (at which ART is administered) and socioeconomic factors. Stopping or suspending ART is a competing event: the person undergoing ART may desist (for example, due to health or financial problems), impeding the event of interest.

We use the Fine and Gray model [6, 24] based on the idea of the Sub-distribution Hazard Function (SHF), which was specifically developed to analyze the association between covariates and risk estimates in the presence of competing events.

### Bivariate Probit regression

The probabilities of being treated with ART in a private or public clinic can be estimated separately using a discrete choice model. However, these models may yield inconsistencies or bias. Due to the substitution between the two alternative service providers, public and private, the two equations’ error terms could be correlated. Therefore, the Bivariate Probit model (Biprobit) is the more appropriate estimator [30]. It has been used in health economics research: to study the impact of a personal income tax reform on health insurance demand [47, 55], assess the probability of hospitalized cardiac patients to experience anxiety or depression [26], or examine the influence of public and private coverage on the mortality of HIV patients [8].

Not all determinants of access to ART are exogenous. Disentangling the cause-and-effect relationship between income or education levels and access to ART can be complicated. Better economic conditions may improve access to ART services, especially in a private clinic, either by paying out-of-pocket or private health insurance. However, higher valorization of women’s time is associated with a higher level of personal and household income, and alternatively, with lower levels of lifetime fertility and therefore greater tendency to require ART services. We address this potential endogeneity by jointly estimating the Biprobit model discussed above, with an additional equation for income and education level. We use parents’ (of the respondent) education level as instrumental variables.

The different models were estimated using the conditional mixed process package [49]. It employs the Geweke-Hajivassiliou-Kean algorithm, a simulated maximum likelihood procedure, to obtain parameter estimates.

All regression estimations were carried out using the software package Stata 15.1.^7^ The descriptive analysis, as well as regression models, were estimated using the SVY survey data module. We controlled for the standard sociodemographic factors and the Autonomous Communities. In our analysis, 10, 5, and 1% were used as statistical significance levels.

## Results

As shown in Table 2, in both samples, the share of women who approached ART services in private clinics is considerably larger than the proportion of those who approached public clinics.

**Table 2:** Variable definitions and descriptive statistics

	Sample for competing risk model: Do public ART services in Spain provide an effective solution compared to private ART services?	Sample for bivariate probit model*: How socioeconomic determinants affect access to ART in public and private clinics in Spain?
Variable	Mean (Linearized standard error) or %	Mean (Linearized standard error) or %
Got pregnant	46.48%	–
Stopped ART treatment	42.73%	–
Currently under ART treatment	10.79%	–
No. of months undergoing ART treatment	21.70 (1.01)	–
Age (years) at onset of ART treatment	33.98 (0.24)	34.51 (0.28)**
Age (years)	41.26 (0.31)	46.82 (0.19)
ART in the private clinic (1 yes; 0 no)	48.86%	38.84%
ART in the public clinic (1 yes; 0 no)	35.29%	21.43%
ART in both public and private clinics (1 yes; 0 no)	15.85%	14.73%
Did not access ART (1 yes; 0 no)	–	25.00%
Marital status single (1 yes; 0 no)	20.41%	18.18%
Education level		
Primary education (reference)	7.90%	12.71%
Secondary education	19.97%	25.41%
Post-secondary education	26.09%	29.31%
High education	46.04%	32.57%
Equivalent household monthly income (€)	1259 (28.85)	1194.29 (28.08)
Personal monthly income (€)	1097.46	1042.60 (36.56)
Number of observations	667	672
Population size	530,619	516,609

Notes: Descriptive statistics are weighted to correct for the complex survey design by the SFS

*Sample include only women aged from 41 to 55

** Mean age at onset of ART treatment for 504 respondent who accessed ART

### Competing risk model

In our sample, 310 women became pregnant, 285 stopped treatment, and 72 were censored (were still ongoing treatment while replying). Moreover, success rates were 52.9% for those treated exclusively in private clinics, compared with 42.4% for public clinics and 40.4% for both public and private.

Table 3 summarizes four competing risk models all based on the respondents’ age at treatment and the clinic type. Models 1.2–1.4 incorporate education, equivalent household monthly income, and personal monthly income, respectively.

**Table 3:** Competing risk models to identify factors associated with success (got pregnant)

Got pregnant (event of interest): 310 Stopping ART treatment (competing event): 285 Lost to follow-up (censored): 72				
Covariates	Model 1.1 SHR (Robust. Std. Err)	Model 1.2 SHR (Robust. Std. Err)	Model 1.3 SHR (Robust. Std. Err)	Model 1.4 SHR (Robust. Std. Err)
Age (years) of access to ART treatment	0.9610 (0.0148)***	0.9520 (0.0183)**	0.9607 (0.0156)**	0.9582 (0.0162)**
ART in the public clinic only (1 yes; 0 no) (reference)				
ART in the private clinic only (1 yes; 0 no)	1.6675 (0.2325)***	1.4573 (0.1640)***	1.6624 (0.2462)***	1.6131 (0.2298)***
ART in both public and private clinics (1 yes; 0 no)	1.1289 (0.2107)	1.9972 (0.1644)	1.1274 (0.2126)	1.1057 (0.2086)
Education level Primary education (reference)				
Secondary education		0.7944 (0.2053)		
Post-secondary education		0.8808 (0.1318)		
High education		1.4136 (0.2415)**		
Equivalent household monthly income (€)			1.0000 (< 0.0001)	
Personal monthly income (€)				1.0000 (< 0.0001)
Akaike’s information criterion (AIC)	3648.78	3638.19	3650.76	3649.10
Number of observations	667	667	667	667

Note: *** *p* < 0.001; ** *p* < 0.01; * *p* < 0.05. *SHR* estimated sub-hazard ratios. Robust standard errors in parenthesis. Robust standard Error are clustered by Autonomous Communities

In the first model, the estimated sub-hazard ratio for patients’ age at ART onset is lower than 1 (0.961). We may interpret this as evidence that a 1-year increase in age is associated with a 3.9% decrease in the incidence of becoming pregnant for women who are still ongoing treatment. Moreover, the estimated sub-hazard ratio for a private clinic is greater than 1 (1.6675), meaning that receiving ART treatment in private against public clinic is associated with a higher incidence of pregnancy controlling regarding age at accessing ART.

Furthermore, the effects of primary and secondary education (model 1.2), equivalent household income (model 1.3), and personal income (model 1.4) were statistically non-significant. Nevertheless, according to the AIC, the best-fit is model 1.2 (lowest value, AIC = 3638.19), where the sub-distribution hazard of becoming pregnant was 41.36% higher for women with high education than for women with primary education.

**Fig. 1:**
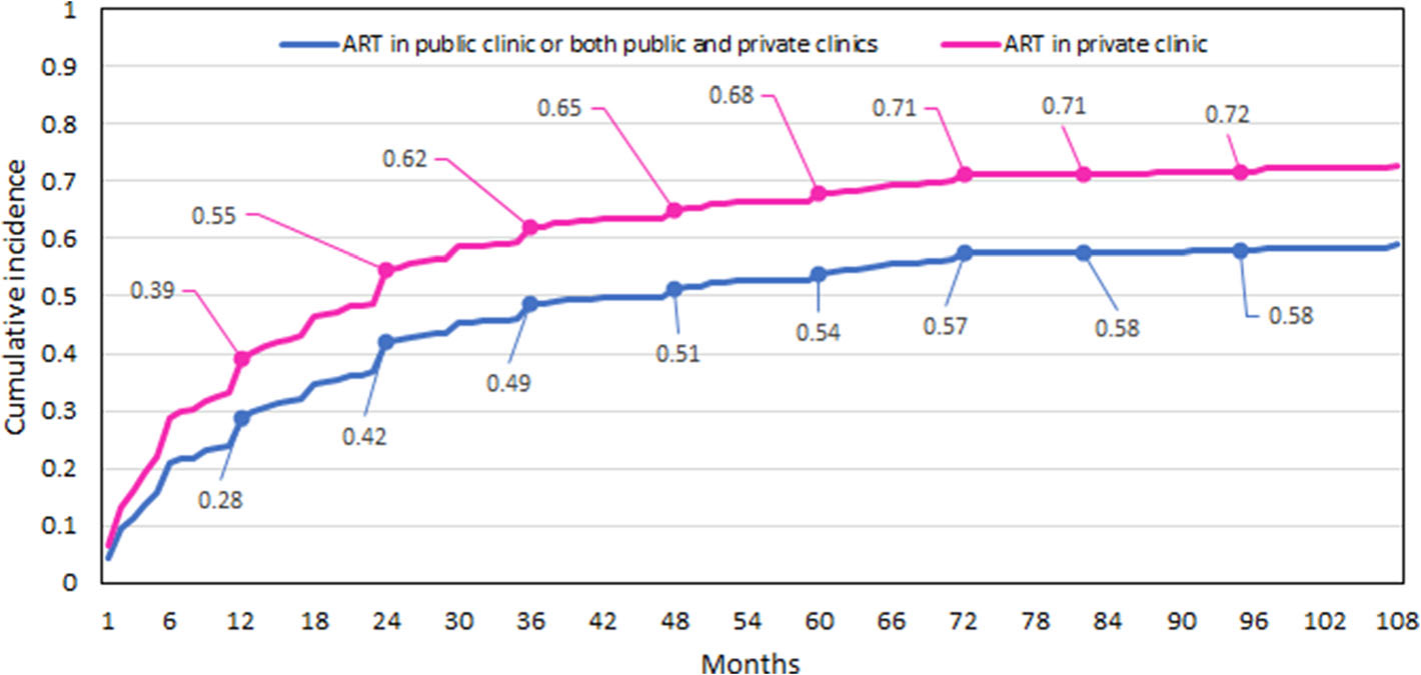
Cumulative incidence function

In our analysis (Table 3), we found that while accessing ART in a private clinic, there is an increase in the incidence of success at the expense of stopping treatment. To demonstrate it visually, we compare two CIF curves based on model 1.2 results (Fig. 1). One for private clinic = 1 and one for private clinic = 0 (ART in a public clinic or both). For both, we adopt the sample’s mean of 34 years as the age at accessing ART and high education level, which counts for 46% of the sample.

Hence, considering high education and 34 years, the cumulative incidence of becoming pregnant within 1, 2, …, and 8 years of treatment is roughly 39, 55%, …, 72% when ART is conducted in private clinics and roughly 28, 42%, …, 58% when ART is conducted in public clinics or both types. Both probabilities consider stopping ART as an alternative.

Figure 1 shows that a gap regarding the average cumulative incidences of success is opening during the first years in favor of being treated exclusively in private clinics. This gap is later not narrowing and remains around 0.14 points.

### Bivariate Probit models for private or public clinics

Table 4 describes the sample analyzed by the Bivariate Probit models:

**Table 4:** Descriptive statistics

	No. of respondents	Mean age at onset	% Singles	High education
Private clinics	261	35.51	16.5%	59.52% [52.99–65.73%] (95% IC)
Public clinics	144	33.40	10.5%	25.76% [19.90–32.63%]
Both types	99	33.07	12%	49.64% [39.50–59.81%]
No access	168		24.5%	

Table 5 presents Bivariate Probit models of two outcomes, ART treatment in private and in public clinics. The estimated correlation coefficients *ρ*_12_ between the random error terms of ART in a private clinic and public clinic equations are ≈ − 0.33, and significant in all models, which denotes a close relationship between the unobserved determinants of the choice of two alternatives.

Household and personal income affect the likelihood of being treated, positively for private clinics and negatively for public ones. Also, a negative association between access to a public clinic and being single is observed. Moreover, with a higher education level, the likelihood of being treated in a private clinic increases and the likelihood of being treated by public clinic decreases.

As we may have expected, parents’ education has the strongest association with the respondent’s education level (mother more than father), followed by personal and household income. All estimations control for the region of residence (results are not shown for simplification).

**Table 5:** Bivariate Probit models: Mixed-process regression

	Model 2.1	Model 2.2	Model 2.3
Private sector ART Eq. 1			
Equivalent household monthly income (€)	0.0007 (0.0001)***		
Personal monthly income (€)		0.0005 (0.001)***	
Marital status single	−0.139 (0.1540)	− 0.103 (0.152)	− 0.2196 (0.1564)
Education level			
Primary education (ref.)			
Secondary education			0.3202 (0.2223)
Post-secondary education			0.7037 (0.2344)***
High education			1.4583 (0.2668)***
Public sector ART Eq. 2			
Equivalent household monthly income (€)	−0.0003 (0.0001)**		
Personal monthly income (€)		−0.0002 (0.0001)**	
Marital status single	−0.3823 (0.1667)**	−0.394 (0.166)**	− 0.3483 (0.1670)**
Education level			
Primary education (ref.)			
Secondary education			−0.4406 (0.2150)**
Post-secondary education			−0.3869 (0.2311)*
High education			−0.7700 (0.254)***
	Household income Equation 3	Personal income Eq. 3	Personal education level Eq. 3
Parents’ education level			
Father			
Primary education (ref.)			
Secondary education	23.79 (124.27)	−10.69 (155.57)	0.1643 (0.1938)
Post-secondary education	134.04 (126.20)	280.19 (190.43)	0.7018 (0.2862)**
High education	313.25 (143.49)**	373.61 (202.39)*	0.5717 (0.2504)**
Mother			
Primary education (ref.)			
Secondary education	315.21 (197.34)	486.24 (198.1)**	0.5141 (0.2266)**
Post-secondary education	319.75 (156.82)**	267.97 (163.57)	0.6806 (0.2329)***
High education	133.84 (117.03)	424.46 (222.4)*	0.7158 (0.3154)**
Number of observations	662	662	662
F-test of overall significance			
F(6, 656) Prob > F	0.0059	0.0001	< 0.0001
ρ12	−0.3285***	−0.3387***	− 0.3354***
ρ13	−0.1885	− 0.2326	−0.2636*
ρ23	0.0048	0.0705	0.0996

Note: *** *p* < 0.001; ** *p* < 0.01; * *p* < 0.05. Robust standard errors in parenthesis. Covariates, not shown here, are regional dummies. ρ12 is the correlation between the error terms in Equation1 and 2; ρ13 is the correlation between the error terms in Eq. 1 and 3; ρ23 is the correlation between the error terms in Eq. 2 and 3

To express the more intuitive change in the predicted probability of accessing private or public clinics, we present the Average Marginal Effects (AME) calculated from the fitted models’ predictions (Table 6) at different values of the remaining covariates. Table 6 contains the AME for all covariates in each of the models.

An increase of 100 euros in the monthly household equivalent income is associated with an increase of 0.022 in the likelihood to access a private clinic and no significant effect in the likelihood to access a public clinic, as well as with a decrease of 0.012 in the probability of failing to access service.^8^

Moreover, an increase of 100 euros in personal income is associated with an increase in the likelihood of accessing private clinics (0.019). It also has a significant negative effect of − 0.008 on the probabilities of both accessing a public clinic and not accessing any service.^9^

Compared with primary education (reference variable), the effect of high education on accessing a private clinic is an average increase of 0.484 points on the probability scale, with a 95% confidence interval of [0.342; 0.626]. Alternatively, (high) education has a negative effect on accessing a public clinic. High education decreases the likelihoods of accessing public clinics or have no access, by 0.254 and 0.177, respectively. Finally, being a single woman decreases the likelihood of accessing public care by 0.115 and increases by 0.134 the overall likelihood of having no access to ART.

**Table 6:** Average marginal effects (AME) for ART in private or public sector

AME on probability of being treated in a private clinic		
	dp/dx	[95% CI]
Equivalent household monthly income (measured in hundreds of euros)	0.02199***	[0.0145; 0.0295]
Personal monthly income (measured in hundreds of euros)	0.01907***	[0.0122; 0.0259]
Primary education (reference)	–	
Secondary education	0.1062	[−0.367; 0.2492]
Post-secondary education	0.2335***	[0.0896; 0.3775]
High education	0.4839***	[0.3423; 0.6255]
Marital status single	−0.0728	[−0.1746; 0.0289]
AME on probability of being treated in a public clinic		
Equivalent household monthly income (measured in hundreds of euros)	−0.00354	[−0.0111; 0.004]
Personal monthly income (measured in hundreds of euros)	−0.00778**	[−0.0152; − 0.0003]
Primary education (reference)	–	
Secondary education	−0.1453**	[−0.2818; − 0.0087]
Post-secondary education	− 0.1276*	[− 0.2746; 0.0195]
High education	− 0.2539***	[− 0.4099; − 0.0979]
Marital status single	−0.1148**	[− 0.2212; − 0.0085]
AME on probability of having no access to ART		
Equivalent household monthly income (measured in hundreds of euros)	−0.01154***	[− 0.0202; − 0.0029]
Personal monthly income (measured in hundreds of euros)	−0.00817**	[− 0.0152; − 0.0012]
Primary education (reference)	–	
Secondary education	−0.008	[−0.1343; 0.1184]
Post-secondary education	−0.1115	[−0.2489; 0.0260]
High education	−0.1772**	[−0.3307; − 0.0238]
Marital status single	0.1335**	[0.0414; 0.2257]

Note: *** *p* < 0.01; ** *p* < 0.05; * *p* < 0.1. dp/dx show the first derivative of the response variable (in terms of probability) with respect to the covariate, for factor levels is the discrete change from the reference level. Standard errors using in the 95% confidence interval of the effect (95% CI) are obtained by the delta method

## Discussion & conclusions

The analyses raised a few trends among Spanish ART patients’ behavior. First, patients who were treated exclusively in public clinics or who combined treatment in both public and private had, on average and throughout the treatment, a lower cumulative incidence of becoming pregnant compared with patients who were treated exclusively in private clinics (Fig. 1). Second, women with a larger income and higher education levels had a higher likelihood of approaching ART services in private clinics and a lower tendency of approaching public clinics. Third, women of lower socioeconomic backgrounds had a lower likelihood of accessing ART services in general. Additionally, our model quantifies the estimated effect of income on the probability of accessing ART in private or public clinics or having no access. Finally, single women had a lower probability of being treated in public clinics and accessing ART services in general since they were excluded from public services in many autonomous communities [36].

Concerning the gap in the cumulative incidence of getting pregnant (model 1.2, Fig. 1). Some patients exercise their right for public coverage first, whereas others begin treatment in a private clinic while remaining on the public clinic’s waiting list [13, 48]. However, public coverage is limited to three cycles and the average waiting time between cycles is around one year. We can conclude that, following 36 months, most patients are treated in private clinics, although the gap remains constant. Based on the National Activity Registry by the Spanish Fertility Society (SEF) and as analyzed by Romero et al. [48], we may assume that the quality of care and success rates in public ART clinics in Spain does not fall from the sector’s standards [48, 52]. It is difficult to determine that private clinics produce a significantly better outcome [13], as they usually handle better prognosis. Moreover, they can treat “good” patients with their own eggs and patients of poor embryonic development or low ovarian reserve with donor eggs.

Hence, we identify three factors that may explain the gap in the cumulative incidence of success. First, long waiting periods in public clinics [40, 25] prolong the treatment and delay success. Second, a patient older than 35 years with a poor prognosis would more commonly be offered to repeat another cycle, using her own eggs, while private centers offer egg donation earlier. Third, some patients would stop treatment after exhausting their right for three subsidized cycles.

As we may learn from the Biprobit models, Spanish patients are aware of public ART disadvantages and perceive better odds within private clinics. Despite the availability of three publicly covered cycles, 41% of the respondent (55% of the patients) accessed ART exclusively in private clinics. At the onset of treatment, their mean age was 35.5, within the eligible age for public care (we have no information about marital status at treatment onset). The tendency to approach private care is due to long waiting lists and other preferences [13, 54]. Only 12% of the respondents combined both types of services. Additionally, 21% accessed only public clinics; of them, 58% did not get pregnant and stopped treatment.

Also, 26% of the respondents required ART but did not access any service. The main barriers are clearly financial, while 21% reported ineligibility for public care, which could be due to advanced age or being single (we do not know their age or status at the time of requiring ART).

Based on these findings, we raise some doubts about the operating principles of public ART services in Spain. The Spanish law 14/2006 on ART is set to provide ART services until 40 years. However, in their current format, public clinics provide an incomplete solution, affected by long waiting lists throughout the treatment, which may interrupt treatments, waste valuable time in their race against aging and could result in reduced outcomes, increased frustration, stress and mistrust [13, 39, 40, 48, 56].

This situation incentivizes most patients, particularly those of higher education and income, to seek services exclusively in private clinics. The dependency on the private sector intensifies ART’s commodification, results in supplier-induced demand, i.e., private centers often rush patients to acquire costly solutions and offer some excessive add-ons [3, 4, 17, 27, 44].

Ideally, public clinics’ capacity should be increased to reduce the gap and meet the law’s intentions. Nevertheless, bearing in mind the limited resources and different priorities in public healthcare, it is arguably suggested that Spanish policymakers would analyze resource allocation optimization in ART, focusing on public clinics’ efficacy by reconsidering both the age limit and the number of cycles. Perhaps, reducing the age of eligibility to public care to below 40 years (for women) may enable faster provision for younger, more disadvantaged patients who suffer from pre-existing rather than age-related infertility and tend to have fewer financial resources [19]. It might also enable providing more than three cycles, which may not be sufficient to fulfill ART potential.

More hypothetically, by funding three ART cycles up to 40 years, this policy signals two wrong messages to the public. First, 40 is still a reasonable age to have a first child and second, three ART cycles should be sufficient. ART registries from various countries, including Spain, illustrate that treatment is becoming less effective after 35 years and that often more cycles are required [14, 53]. Moreover, it has been suggested that public coverage may lead to an increase in average age at first birth and might even negatively affect fertility rates [29, 37]. Therefore, ART’s limitations and risks in advanced maternal age should also be considered, alongside alternatives that may reduce infertility [34] to avoid a social trend of parenthood postponement [35].

Our study has a few limitations, mainly derived from the available information by the SFS and from the fact that the survey was not designed to directly answer our research questions. It enables us to conduct a cross-section analysis since the participants were surveyed only once at a particular moment. More precisely, the SFS provides data about income and marital status at the time of replying and not while attending or requiring services. Conversely, a longitudinal study could provide clearer conclusions. It is reflected mainly by the variation in income and marital status, which may change during a person’s life-course, unlike education levels, which mostly maintain constant. Moreover, the SFS does not report about insurance, waiting periods and the number of children. It reports both employment status and urban status, but we assume that household and personal income already reflect those (and insurance). Additionally, autonomous communities may reflect urban status, as well as distance from clinics.

Finally, we recommend several research directions that can contribute to better understanding and policy planning. First, it would be interesting from a public perspective to evaluate public ART provision in Spain by operational analysis to help find a more effective and efficient allocation of the limited resources. Second, a model of (partially) public funding and private provision (such as the “PADI” plan for dental health program for children [10]) should be evaluated and considered as an alternative, despite that such model may create conflict of interests between private enterprises and policy makers, lead to supplier-induced demand, and may enhance the principal-agent problem. Fourth, considering that ART in private clinics may be costly, conducting a survey or a series of interviews could raise important insight into young couples’ ongoing financial burden. Finally, about the SFS (conducted in 1999 and 2008), conducting a public fertility survey requires a significant public investment and aims to provide potential researchers with valuable data. When the opportunity to conduct a subsequent survey emerges, its designers should elaborate on an open discussion with researchers who may find interest in exploring the collected data. Sharing experiences and common interests may assist in directing the survey towards researchers’ needs.

## Data Availability

https://www.ine.es/dyngs/INEbase/es/operacion.htm?c=Estadistica_C&cid=1254736177006&menu=resultados&idp=1254735573002#!tabs-1254736195659

## Appendix

### Summary of Equations

**1st model:**

The SHF for event *p* (get pregnant) can be expressed as:

1 $$ {h}_p(t)=\underset{\Delta  \to 0}{\lim}\frac{\Pr \left(t<{T}_p<t+\Delta  t|{T}_p>t\cup {T}_{p^{\prime }}\le t,{p}^{\prime}\ne p\right)}{\Delta  t} $$

The above function estimates the hazard rate for event type *p*, at time *t*, based on the risk set that remains at time *t* after accounting for all previously occurring event types, including competing events. The cumulative incidence function (CIF) based proportional hazard model is then defined as:

2 $$ {CIF}_p(t)=1-\exp \left(-\mathit{\exp}\left( X\beta \right){\int}_0^t{h}_p(t) dt\right) $$

We can interpret the betas as we do for the betas estimated from a Cox model, except that the competing risk model estimates the effect of certain covariates in the presence of competing events. The exponential of beta coefficients represents the sub-hazard ratios. A coefficient greater than 1 means that increasing that covariate is associated with a higher incidence of success (getting pregnant). The Sub-distribution hazard ratios also describe the relative effect of covariates on the sub-distribution hazard function. Therefore, the covariates may be interpreted as having an effect on the cumulative incidence function or the probability of events occurring over time [6].

**2nd Model:**

The system of equations to be estimated is:

3 $$ {\displaystyle \begin{array}{c}{y}_{1i}^{\ast }={X}_i{\beta}_1+{u}_{1i}\\ {}{y}_{2i}^{\ast }={X}_i{\beta}_2+{u}_{2i}\end{array}} $$

For individual *i*, *i* = 1, … *N*, where *y**_*1*_ and *y**_*2*_, are unobserved latent variables indicating the propensity of being treated by ART in private or public clinics, respectively. *X*_*i*_ denotes the vector of covariates. The remaining terms in (3) are the vectors of parameters to be estimated *β*_1_ and *β*_2_; the random error terms, *u*_1*i*_ and *u*_2*i*_, where (*u*_1*i*_, *u*_2*i*_)~*BVN*(0, 0, 1, 1, *ρ*); and the correlation coefficient, *ρ* = *corr*(*u*_1*i*_, *u*_2*i*_).

## Footnotes

1 We refer here to marriage or domestic partnership.

2 Partially revised by Law 19/2015

3 Public clinics provide donor eggs only in exceptional cases of genetic disorders, or early ovarian failure before the age of 35

4 https://www.ine.es/en/prensa/ef_2018_d_en.pdf

5 The Spanish law: RD 1030/2006 enables access to public coverage of ART to single women (and lesbian couples). However, between 2013 and 2019, the health ministry restricted public ART services exclusively to married couples, despite that in some autonomous communities, singles and lesbian couples were still eligible.

6 The Oxford equivalence scale (OECD, 1982) is used to adjust the predicted disposable monthly income for differences in household composition.

7 StataCorp. (2017). Stata statistical software: Release 10. College Station, TX: StataCorp LP

8 Concerning the third part of Table 6 (no access to ART), it is important to note that a negative effect on the probability of not accessing ART is interpreted as a positive effect on the probability of accessing ART in general. The AME on the probability of having no access to ART was estimated separately based on a new recursive mixed process model with two equations: first, an equation using an OLS model for income or ordered Probit for education level respectively, and second, an equation with a Probit model estimating the probability of having no access to ART.

9 See the previous note.
